# Supplementary material for: Content-rich biological network constructed by mining PubMed abstracts
Source: BMC Bioinformatics. 2004 Oct 8;5:147. doi: 10.1186/1471-2105-5-147 (PMC528731; doi:10.1186/1471-2105-5-147)
Supplement: Additional File 2 — The original results of the above study (non-essential files are deleted to keep the file size under the limit set by BMC bioinformatics). [file 1471-2105-5-147-S2.bz2 › chilibotAdditionalFile2/dip05/220ID7877691E730/html/RB1_UBTF.html]

 


 **RB1** and **UBTF** 
  
Found 4 abstracts in PubMed, retrieved 4.  
 

 What does Google say? 
 PDF only 
| .edu only 

---

**Interactive relationship** (e.g. stimulation, inhibition, etc)

**Non-interactive relationship** (e.g. studied together, co-existance, homology, etc.)

- We have previously demonstrated that the protein encoded by the retinoblastoma  [ **RB1** ]  susceptibility gene Rb functions as a regulator of transcription by RNA polymerase I rDNA transcription by inhibiting UBF  [ **UBTF** ]  mediated transcription.  Ref: 11042686 Oncogene, 2000
